# Demethylzeylasteral Exhibits Strong Inhibition towards UDP-Glucuronosyltransferase (UGT) 1A6 and 2B7

**DOI:** 10.3390/molecules17089469

**Published:** 2012-08-08

**Authors:** Jin-Wen Zhao, Gui-Hua Wang, Min Chen, Lian-Hua Cheng, Xiao-Qi Ji

**Affiliations:** Department of Nephrology, Huai’an First People’s Hospital, Nanjing Medical University, 6 Beijing Road West, Huai’an, Jiangsu 223300, China

**Keywords:** *Tripterygium wilfordii* Hook F., demethylzeylasteral, UDP-glucuronosyl-transferase (UGT)

## Abstract

Inhibition of UDP-glucuronosyltransferase (UGT) isoforms can result in severe clinical results, including clinical drug-drug interactions (DDI) and metabolic disorders of endogenous substances. The present study aims to investigate the inhibition of demethylzeylasteral (an important active component isolated from *Tripterygium wilfordii* Hook F.) towards three important UGT isoforms UGT1A6, UGT1A9 and UGT2B7. The results showed that 100 μM of demethylzeylasteral exhibited strong inhibition towards UGT1A6 and UGT2B7, with negligible influence towards UGT1A9. Furthermore, Dixon and Lineweaver-Burk plots showed the inhibition of UGT1A6 and UGT2B7 by demethylzeylasteral was best fit to competitive inhibition, and the inhibition kinetic parameters (Ki) were calculated to be 0.6 μM and 17.3 μM for UGT1A6 and UGT2B7, respectively. This kind of inhibitory effect need much attention when demethylzeylasteral and demethylzeyasteral-containing herbs (e.g., *Tripterygium wilfordii* Hook F.) were co-administered with the drugs mainly undergoing UGT1A6, UGT2B7-catalyzed metabolism. However, when extrapolating the *in vivo* clinical results using our present *in vitro* data, many complex factors might affect final results, including the contribution of UGT1A6 and UGT2B7 to the metabolism of compounds, and the herbal or patients’ factors affecting the *in vivo* concentration of demethylzeylasteral.

## 1. Introduction

**Figure 1:**
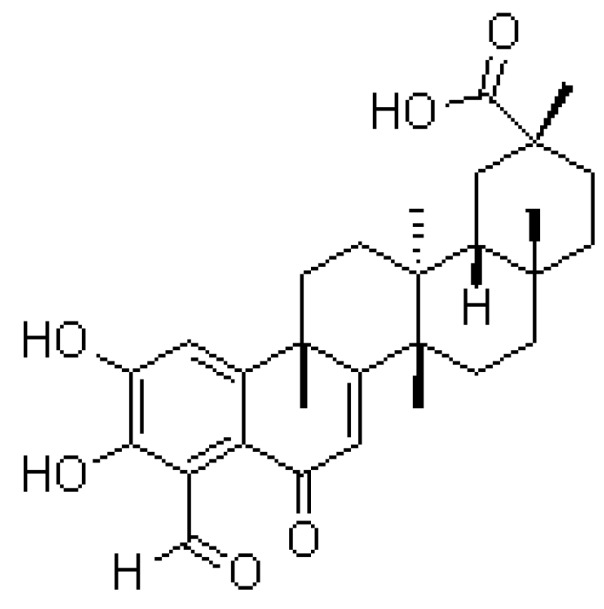
The structure of demethylzeylasteral.

*Tripterygium wilfordii* Hook F., commonly known in China as Lei-Gong-Teng, has been used to treat rheumatoid arthritis, chronic nephritis, ankylosing spondylitis and various skin diseases [1,2,3]. Demethylzeylasteral (Figure 1), the active component isolated from *Tripterygium wilfordii* Hook F., has been demonstrated to exhibit some pharmacological activity, including immunosuppressive effects [4]. 

Herb-drug interactions have become a challenging problem in clinical application of herbs, and one of the major reasons is the influence of herbs on the pharmacokinetic behaviour of drugs [5]. In previous studies, much attention has been paid to the inhibition of cytochrome P450s (CYPs) by herbal constituents. For example, the ginsenosides have been reported to inhibit the multiple CYP isoforms, which is speculated to be a potential reason for a variety of ginseng-drug interactions, including ginseng-loop diuretics, ginseng-phenelzine, ginseng-digoxin, and ginseng-warfarin interactions [6]. Other herbal components exerting inhibition towards CYP isoforms contain the active components isolated from *Ginkgo biloba* [7], flavonoids [8], and Danshen's constituents [9].

UDP-glucuronosyltransferases (UGTs) are the most important enzymes catalyzing the conjugation of various xenobiotics and endogenous substances, which accounts for >35% of all phase II drug metabolism [10]. Many important endogenous substances and xenobiotics have been conjugated by UGTs, such as bilirubin, steroid hormones, thyroid hormones, bile acids, and fat-soluble vitamins [11,12]. Inhibition of UGTs-catalyzed glucuronidation might induce severe clinical results, including drug-drug interactions and metabolic disorders of endogenous substances. Therefore, evaluation of inhibitory capability of compounds towards UGT isoforms has become a routine task of pharmacy industry.

For investigation of compounds’ inhibition towards CYP isoforms, many specific probe substrates and human liver microsomes have been developed. In contrast, few specific probe substrates exist for UGT isoforms. Therefore, when evaluating the inhibition of compounds towards UGT isoforms, the non-specific substrate 4-MU was the routine probe, and the recombinant UGT isoforms were commonly used as enzyme sources [13,14].

The aim of the present study is to investigate the inhibition of demethylzeylasteral towards three important UGT isoforms UGT1A6, UGT1A9 and UGT2B7. Furthermore, the detailed inhibition kinetic type and parameters were determined.

## 2. Results and Discussion

**Figure 2:**
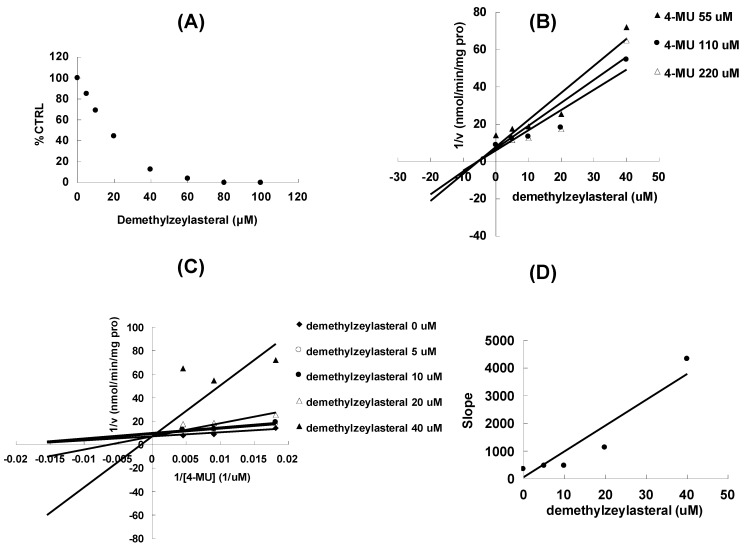
Inhibition kinetic analysis of demethylzeylasteral towards recombinant UGT1A6-catalyzed 4-MU glucuronidation. (**A**) Demethylzeylasteral showed dose-dependent inhibition towards UGT1A6; (**B**) Dixon plot of demethylzeylasteral inhibition towards UGT1A6-catalyzed 4-MU glucuronidation; (**C**) Lineweaver-Burk plot of demethylzeylasteral inhibition towards UGT1A6-catalyzed 4-MU glucuronidation; (**D**) Second plot using slope (obtained from Lineweaver-Burk plot) *vs.* the concentration of demethylzeylasteral.

**Figure 3:**
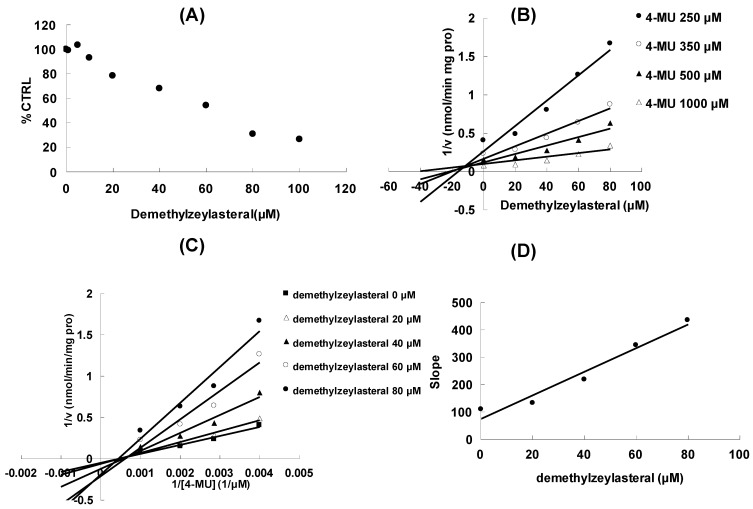
Inhibition kinetic analysis of demethylzeylasteral towards recombinant UGT2B7-catalyzed 4-MU glucuronidation. (**A**) Demethylzeylasteral showed dose-dependent inhibition towards UGT2B7; (**B**) Dixon plot of demethylzeylasteral inhibition towards UGT2B7-catalyzed 4-MU glucuronidation; (**C**) Lineweaver-Burk plot of demethylzeylasteral inhibition towards UGT2B7-catalyzed 4-MU glucuronidation; (**D**) Second plot using slope (obtained from Lineweaver-Burk plot) *vs.* the concentration of demethylzeylasteral.

At 100 μM of demethylzeylasteral, the residual activity of UGT1A6, UGT1A9 and UGT2B7-mediated 4-MU glucuronidation was 0.8 ± 0.1%, 61.3 ± 5.6% and 14.8 ± 1.1% of control group. Furthermore, inhibition kinetic analysis was carried out to determine the inhibition type and kinetic parameters for UGT1A6 and UGT2B7, which activities were inhibited by more than 50% at a concentration of 100 μM. The results showed that demethylzeylasteral exhibited a concentration-dependent inhibitory behaviour towards UGT1A6 (Figure 2A) and UGT2B7-catalyzed 4-MU glucuronidation (Figure 3A), with the IC_50_ values of 15.2 ± 0.6 μM and 62.5 ± 1.6 μM, respectively.

Dixon (Figure 2B and Figure 3B) and Lineweaver-Burk (Figure 2C and Figure 3C) showed that inhibition of UGT1A6 and UGT2B7 by demethylzeylasteral was best fit to competitive inhibition. Second plot (Figure 2D and Figure 3D) using slope (obtained from Lineweaver-Burk plot) *vs.* the concentration of demethylzeylasteral was used to determine the inhibition kinetic parameters (Ki), and the values were calculated to be 0.6 μM and 17.3 μM for UGT1A6 and UGT2B7, respectively. These results were consistent with the previous literature in which another active component of *Tripterygium wilfordii* Hook F. celastrol (with the similar structure with demethylzeylasteral) also exhibited strong inhibition towards UGT1A6 and UGT2B7 [13].

UGT1A6 is involved in the glucuronidation of various drugs, toxins, and endogenous substrates, including acetaminophen, benzopyrene, and serotonin [15,16]. The factors influencing the UGT1A6 activity might induce the important pharmacological, toxicological and physiological consequences. Previous reports have showed that 120-fold variability of UGT1A6 activity in human liver induce the 13-fold difference for glucuronidation of serotonin [16]. UGT2B7 has been regarded as one of the most important UGT isoforms, and can participate in the glucuronidation of various compounds, including various steroid hormones (androsterone, epitestosterone), fatty acid, carboxylic nonsteroidal anti-inflammatory drugs, and anticarcinogens (all-*trans* retinoic acid) [17,18].

Taken together, demethylzeylasteral showed strong inhibition towards UGT1A6 and UGT2B7, with negligible influence towards UGT1A9. This kind of inhibitory effect needs much attention when demethylzeylasteral and demethylzeyasteral-containing herbs are co-administered with the drugs mainly undergoing UGT1A6, UGT2B7-catalyzed metabolism. However, when extrapolating the *in vivo* clinical results using our present *in vitro* data, many complex factors might affect final results, including the contribution of UGT1A6 and UGT2B7 to the metabolism of compounds, and the herbal or patients’ factors affecting the *in vivo* concentration of demethylzeylasteral. It should be noted that the inhibition of demethylzeylasteral towards other UGT isoforms are not be included in this study, and the future experiments will be performed to examine this factor.

## 3. Experimental

### 3.1. Chemicals and Reagents

Demethylzeylasteral with purity ≥98% was obtained from Weikeqi Biotechnology Co. Ltd. (Sichuan, China). 4-Methylumbelliferone (4-MU), 4-methylumbelliferone-β-D-glucuronide (4-MUG), Tris-HCl, 7-hydroxycoumarin and uridine 5′-diphosphoglucuronic acid (UDPGA) (trisodium salt) were purchased from Sigma-Aldrich (St. Louis, MO, USA). Recombinant UGT1A6, 1A9 and UGT2B7 expressed in baculovirus were obtained from BD Gentest Corp. (Woburn, MA, USA). All other reagents were of HPLC grade or of the highest grade commercially available.

### 3.2. Incubation and Analysis Conditions for Enzyme Inhibition Experiment

4-MU, the nonspecific probe substrate for UGT isoforms, was employed to investigate the inhibition of UGT1A6, UGT1A9 and UGT2B7 by demethylzeylasteral. The mixture (200 μL total volume) contained recombinant UGTs (final concentration: 0.025, 0.05 and 0.05 mg/mL for UGT1A6, UGT1A9 and UGT2B7) 5 mM UDPGA, 5 mM MgCl_2_, 50 mM Tris-HCl buffer (pH 7.4), and 4-MU in the absence or presence of different concentrations of demethylzeylasteral. The concentrations of 4-MU were as follows: 110 μM for UGT1A6, 30 μM for UGT1A9 and 350 μM for UGT2B7. Demethylzeylasteral was dissolved in methanol and the final concentration of methanol was 0.5% (v/v). After 5 min pre-incubation at 37 °C, the UDPGA was added to the mixture to initiate the reaction. Incubation time was 120 min for UGT2B7, and 30 min for UGT1A6 and UGT1A9, respectively. The reactions were quenched by adding 100 μL acetonitrile with 7-hydroxycoumarin (100 μM) as internal standard. The mixture was centrifuged at 20,000 ×g for 10 min and an aliquot of supernatant was transferred to an auto-injector vial for HPLC analysis.

The HPLC system (Shimadzu, Kyoto, Japan) containing a SCL-10A system controller, two LC-10AT pumps, a SIL-10A auto injector, and a SPD-10AVP UV detector was used in the present study. C18 column (4.6 × 200 mm, 5 µm, Kromasil) was employed to seperate the substrate and metabolites at a flow rate of 1 mL/min with UV detection at 316 nm. The mobile phase consisted of H_2_O containing 0.5% (v/v) formic acid (A) and acetonitrile (B). The following gradient conditions were used: 0–15 min, 95–40% A; 15–20 min, 10% A; 20–30 min, 95% A. To determine the inhibition kinetic type and calculate the inhibition parameters, various concentrations of demethylzeylasteral were added to reaction mixtures containing different concentrations of 4-MU. Dixon and Lineweaver plots were adapted to determine the inhibition type, and the second plot of slopes from Lineweaver-Burk plot *vs.* demethylzeylasteral concentrations was utilized to calculate Ki value.

## 4. Conclusions

Among the tested UGT isoforms, UGT1A6 and UGT2B7 were inhibited by demethylzeylasteral, which indicated the necessity to monitor the potential clinical interactions between dimethyl- zeylasteral-containing herbs and clinical drugs mainly undergoing UGT1A6, UGT2B7-catalyzed metabolism.
